# The evolution of phase constitution and microstructure in iron-rich 2:17-type Sm-Co magnets with high magnetic performance

**DOI:** 10.1038/s41598-018-27487-x

**Published:** 2018-06-14

**Authors:** Chaoyue Zhang, Zhuang Liu, Ming Li, Lei Liu, Tianyi Li, Renjie Chen, Don Lee, Aru Yan

**Affiliations:** 1 Key Laboratory of Magnetic Materials and Devices, Ningbo Institute of Material Technology and Engineering, CAS, Ningbo, 315201 China; 2 University of Chinese Academy of Sciences, Beijing, 100049 China; 3 University of Dayton, Dayton, OH USA

## Abstract

Iron-rich 2:17-type Sm-Co magnets are important for their potential to achieve high coercivity and maximum magnetic energy product. But the evolution of phase structure, which determines magnetic properties, remains an unsolved issue. In this study, the phase constitution and microstructure of solution-treated 2:17-type Sm-Co alloys are studied. The increase of Fe content promotes the ordering transformation from the 1:7H phase to partially ordered 2:17R and lamellar Zr-rich 1:3R phase. This ordering transformation is mainly due to the competitive atoms occupation of Zr, Fe and Sm in the 1:7H phase. To ease this competition, we modify Sm content in iron-rich 2:17-type Sm-Co magnets. Different solution precursors and corresponding cellular structures are observed. Solution precursor with 1:7H, partially ordered 2:17R, 2:17H and 1:3R phase evolves into uneven and incomplete cellular structures, while solution precursor with partially ordered 2:17R phase forms larger cell size with less lamellar phase, thus poor coercivity and magnetic energy product. However, solution precursors with single 1:7H phase evolve into uniform cellular structures and perform high coercivity and magnetic energy product. Our results indicate if a single 1:7H phase could be obtained in solution-treated 2:17-type Sm-Co magnets with higher iron content, much higher magnetic properties could be achieved.

## Introduction

Highly heat-resistant permanent magnets with high-energy density are widely required in microwave tubes, gyroscope and accelerometer, magnetic bearings, sensors, and high-efficiency motors. Sm(Co,Fe,Cu,Zr)_z_ magnet is ideal for these applications because of its excellent temperature tolerance, high corrosion resistance and Dy/Tb elements free^1,2^. Because of high price of Dy/Tb, Pr/Nd and worse temperature stability issues found in Nd-Fe-B magnets, Sm(Co,Fe,Cu,Zr)_z_ magnets can be extremely viable alternative for these applications with operating temperatures above 180 °C. With the magnetic energy product of Sm(Co,Fe,Cu,Zr)_z_ magnets further increasing, the Nd-Fe-B magnets applied above 150 °C could also be replaced by Sm(Co,Fe,Cu,Zr)_z_ magnets. The saturation magnetization and remanence of Sm(Co,Fe,Cu,Zr)_z_ magnet, which increase rapidly with the increase of iron content, is mostly determined by Sm_2_(Co_1−x_Fe_x_)_17_ matrix phase. Furthermore, the Sm_2_(Co_1−x_Fe_x_)_17_ compounds retain the easy axis magnetic orientation for x up to 0.5^3^. The estimated theoretical limits of maximum energy product *(BH)*_max_ for Sm_2_(Co_1−x_Fe_x_)_17_ reach a peak of over 60*MGsOe* for x = 0.4^4,5^. Thus, the addition of iron is an effective method to improve the saturation magnetization of Sm(Co,Fe,Cu,Zr)_z_ magnets^6,7^, which is a promising route to further improve the magnetic energy product. However, iron-rich Sm(Co,Fe,Cu,Zr)_z_ magnets tend to form a coarse and inhomogeneous nanoscale structure^8,9^, which results in a low coercivity and poor squareness of the demagnetization curve.

As for the 2:17-type Sm-Co sintered magnet, the cell-like structure (described as cellular structure) plays a critical role in its excellent magnetic properties^10–12^. It is known that the cellular structure is formed by 2:17R cell phase (Th_2_Zn_17_, *R-3m*), 1:5 cell boundary phase (CaCu_5_, *P6/mmm*) and lamellar Zr-rich 1:3R phase precipitation hardening from the solution precursor^13–16^. The single 1:7H precursor phase (TbCu_7_, *P6/mmm*) is critical to obtain the desired final cellular structure^17^. In 2:17 type Sm-Co sintered magnets with low iron content, the precursor with single 1:7H phase is easy to be obtained. As for iron-rich 2:17-type Sm-Co sintered magnet, Tang *et al*.^18^ and Hadjipanayis *et al*.^8^ reported that the addition of a certain amount of Fe is necessary to develop a uniform cellular structure with a larger cell size and higher coercivity, but the excessive addition leads to inhomogeneous microstructure and thus deteriorates the coercivity. Guo *et al*.^19^ reported that the formation of Zr-rich phase and needle-like Fe-Co phase in Sm(Co_bal._Fe_0.27_Cu_0.088_Zr_0.025_)_7.5_ magnet is the main reason for the reduction in magnetic properties, while Horiuchi *et al*.^7^ reported that the precipitation of 2:7 phase prevents the formation of cellular structure during aging treatment in Sm(Co_bal._Fe_0.35_Cu_0.06_Zr_0.018_)_7.8_ magnet. These impurity phases would lead to incomplete cellular structure and decreased magnetic properties. However, the possible reason that the main phase in solution precursor is ignored in iron-rich 2:17-type SmCo magnets. Up to now, the mechanism of phase formation and the microstructure evolution of iron-rich Sm(Co,Fe,Cu,Zr)_z_ magnets are still not clear. In this paper, we present a systematic research on the phase constitution and microstructure of 2:17-type Sm-Co alloys and magnets with high Fe content. We demonstrate that the increasing Fe content exert a complex influence on phase structures of the solution precursors. By control of phase structures in the solution precursors, different cellular structures are observed, which can clearly explain the formation of the inhomogeneous microstructure. Based on the microstructure modulation, both high magnetic coercivity and magnetic energy product in iron-rich 2:17-type Sm-Co magnets are achieved.

## Results

### Phase constitution evolution before and after solution treatment

**Figure 1:**
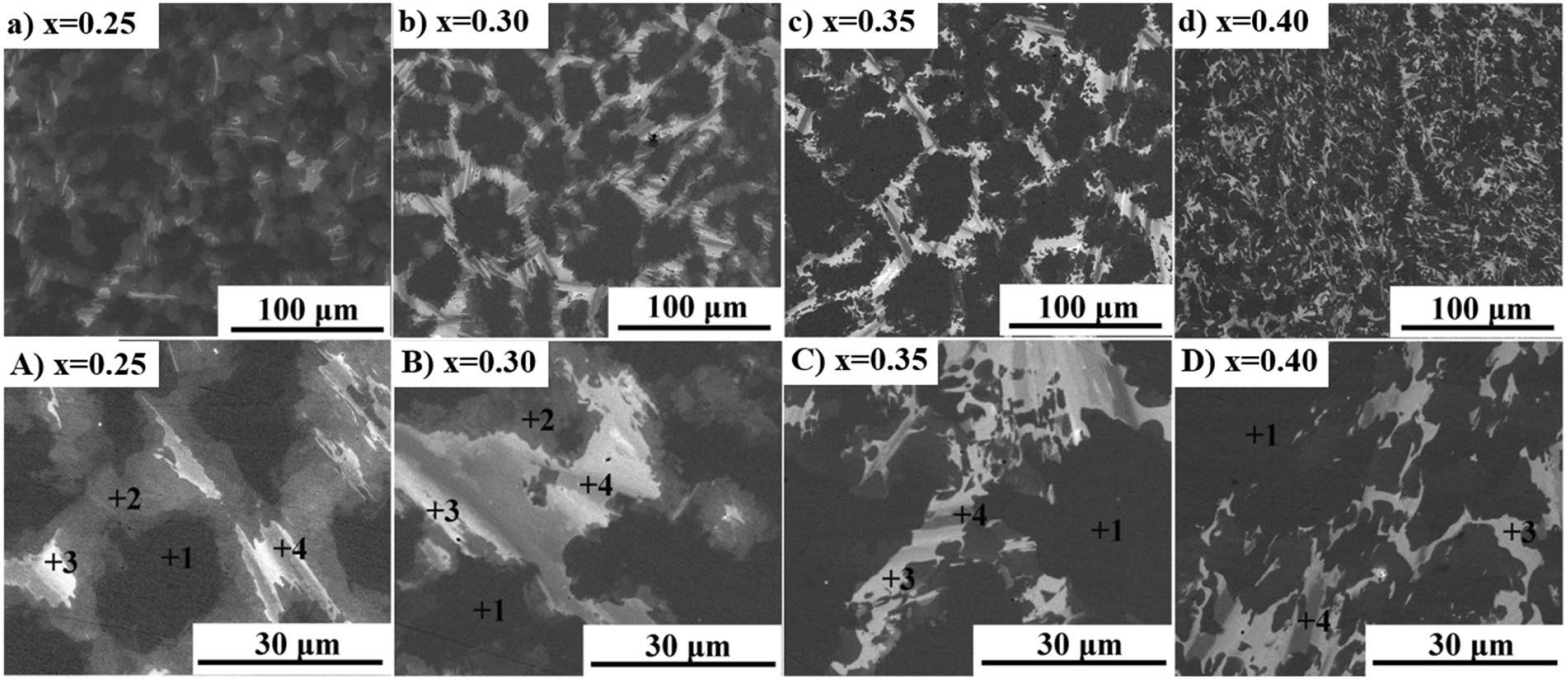
SEM images of Sm(Co_bal._Fe_x_Cu_0.053_Zr_0.02_)_7.84_ as-cast alloys perpendicular to the direction of texture. (**a**) x = 0.25, (**b**) x = 0.30, (**c**) x = 0.35, and (**d**) x = 0.40; (A–D) show broader areas corresponding to the same magnets for (a-d), respectively. Position 1, 2, 3 and 4 represent for 1:7H, Cu-rich 1:5, Cu-rich 2:7 and Zr-rich 1:3, respectively.

Figure 1 shows scanning electron microscope (SEM) images of Sm(Co_bal._Fe_x_Cu_0.053_Zr_0.02_)_7.84_ (x = 0.25, 0.30, 0.35, and 0.40) as-cast alloys. A network structure was observed in alloys with x ≤ 0.35 (Fig. 1a–c), which becomes larger as the Fe content increases. When x reaches to 0.4 (Fig. 1d), the network structure transforms to a flocculent structure. The detailed composition of these phases in alloys are listed in Table 1. According to the results of EDS, phases marked with position 1, position 2, position 3 and position 4 (show in Fig. 1a–d) are 1:7H matrix phase, Cu-rich 1:5 phase, Cu-rich 2:7 phase and Zr-rich phase, respectively. A similar microstructure feature has also been observed in Sm(Co_bal._Fe_0.237_Cu_0.051_Zr_0.034_)_7.7_ as-cast alloys^20^ and Sm(Co_bal._Fe_x_Cu_0.08_Zr_0.03_)_8.2_ (x = 0.23, 0.26, and 0.28) as-cast alloys^21^. According to the chemical composition, the Zr-rich phase can be shortened in form of (SmZr)_1_(CoFeCu)_3_, which is in accord with the results of Campos^22^. Furthermore, it can be seen that the content of Zr in Zr-rich 1:3 phase increases with the increase of the Fe content, which indicates that the addition of Fe tends to promote the segregation of Zr in 1:3 phase. The SEM images also show the decreased quantity of Cu-rich 1:5 phases and increased quantity of Cu-rich 2:7 phases with the increase of Fe content.

**Table 1:** Phase composition of as-cast Sm(Co_bal_Fe_x_Cu_0.053_Zr_0.02_)_7.84_ (x = 0.25,0.30,0.35, and 0.40) alloys.

x	Site	Element (at. %)					Phase
		Sm	Co	Fe	Cu	Zr	
0.25	1	10.7	58.9	25.2	3.6	1.6	1:7H
	2	14.6	55.4	20.6	8.3	1.1	Cu-rich 1:5
	3	20.7	49.3	14.7	14.3	1.0	Cu-rich 2:7
	4	13.7	56.6	16.4	4.7	8.6	Zr-rich 1:3
0.30	1	10.2	55.4	30.2	2.9	1.3	1:7H
	2	15.0	50.6	25.2	8.5	0.7	Cu-rich 1:5
	3	21.8	42.0	17.4	18.1	0.7	Cu-rich 2:7
	4	13.4	54.4	18.9	4.4	8.9	Zr-rich 1:3
0.35	1	10.1	51.0	34.7	2.8	1.4	1:7H
	3	21.1	44.4	19.9	12.6	2.0	Cu-rich 2:7
	4	11.4	50.5	22.8	3.5	11.8	Zr-rich 1:3
0.40	1	10.0	46.5	39.2	2.8	1.5	1:7H
	3	17.9	47.7	24.9	6.3	3.2	Cu-rich 2:7
	4	9.6	47.5	25.2	2.7	15.0	Zr-rich 1:3

**Figure 2:**
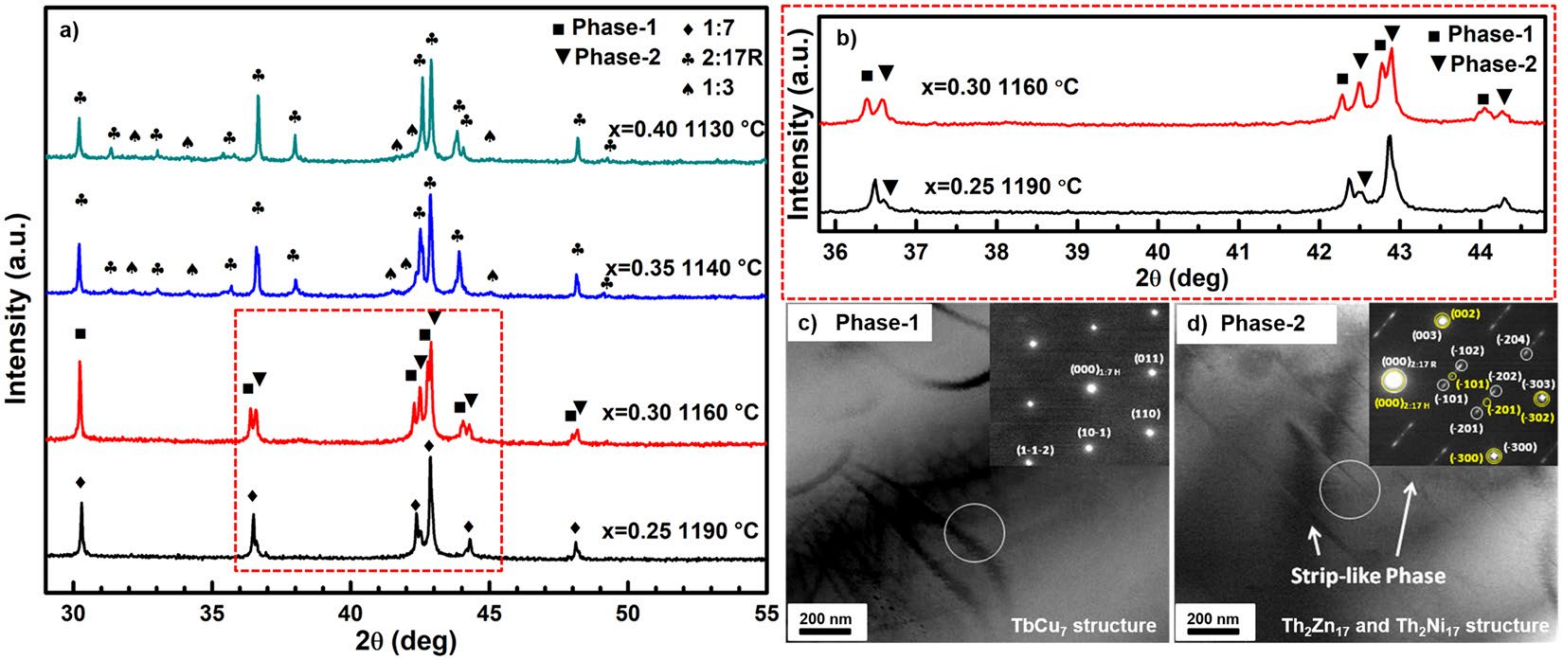
(**a**) XRD patterns of solution treated Sm(Co_bal._Fe_x_Cu_0.053_Zr_0.02_)_7.84_ (x = 0.25, 0.30, 0.35, and 0.40) alloys. (**b**) Magnified XRD patterns of unknown phase (x = 0.25 and 0.30). (**c**,**d**) TEM bright field (TEM-BF) images and the corresponding selected area electron diffraction pattern (SAED) in two different regions of solution treated Sm(Co_bal._Fe_0.30_Cu_0.053_Zr_0.02_)_7.84_ alloys.

Figure 2a shows the X-ray diffraction (XRD) patterns of solution-treated Sm(Co_bal_Fe_x_Cu_0.053_Zr_0.02_)_7.84_ (x = 0.25, 0.30, 0.35, 0.40) alloys. With the increase of Fe content from 0.25 to 0.40, the optimum solution temperature decreases from 1190 °C to 1130 °C. A nearly single 1:7H phase is obtained in solution treated alloy with x = 0.25, except for a minor unknown phase pattern (Fig. 2b). The diffraction peaks of this unknown phase increase and two patterns of main phase (denoted as Phase-1 and Phase-2) are observed in alloy with x = 0.30. Zr-rich 1:3 phase patterns are shown in alloys with x ≥ 0.35. Moreover, the characteristic (2 0 4)_Th2Zn17_ peak of 2:17R phase is observed in alloys with x = 0.35 and increases obviously when x = 0.40, which means that the main phase is unstable and tends to transfer from 1:7H to 2:17R with precipitation of Zr-rich phase in solution-treated alloys as the Fe content increases.

To clarify the different main phase in x = 0.30 solution-treated alloy, its microstructure was investigated by Transmission electron microscope (TEM), as is shown in Fig. 2c,d. The selected area electron diffraction (SAED) pattern of Phase-1 is the superposition diffraction patterns for the ${[{\rm{1}}\bar{1}{\rm{1}}]}_{1:\mathrm{7H}}$ zone axis of 1:7H phase with TbCu_7_ structure (*P6/mmm*). The corresponding SAED pattern of Phase-2 is the superposition diffraction patterns for the [0 1 0]_2:17R_ zone axis of 2:17R phase with Th_2_Zn_17_ structure (*R-3m*) and the [0 1 0]_2:17H_ zone axis of 2:17H phase with Th_2_Ni_17_ structure (*P63-mmc*), along with strip-like phase precipitates. Interestingly, many diffraction spots, e.g. ${(\bar{2}{\rm{0}}{\rm{1}})}_{2:\mathrm{17R}}$, ${(\bar{2}{\rm{0}}{\rm{4}})}_{2:\mathrm{17R}}$, are elongated in Phase-2, which indicates that the corresponding crystal planes have many plane defects, e.g. stacking faults. The plane defects weaken the X-ray diffraction peak of the corresponding crystal plane. Thus (2 0 4)_2:17R_ peaks in solution-treated alloy with x = 0.30 cannot be observed in XRD profiles. As we know, the Th_2_Ni_17_ structure (2:17H phase structure) is a stacking of two types of mixed planes in *…ABABAB…* sequence and the Th_2_Zn_17_ structure (2:17R phase structure) is a stacking of three types of mixed planes in *…ABCABC…* sequence. So partially ordered mixture with Th_2_Zn_17_ structure and Th_2_Ni_17_ structure is formed due to stacking fault. In a word, the Phase-2 can be referred as a partially ordered mixture with Th_2_Zn_17_ structure and Th_2_Ni_17_ structure, while Phase-1 is a completely disordered 1:7H phase with TbCu_7_ structure. This phenomenon is mainly due to the competitive atoms occupation between Zr, Fe and Sm in 1:7H phase, which would be discussed later.

### Effect of Sm content on microstructure and magnetic properties

**Figure 3:**
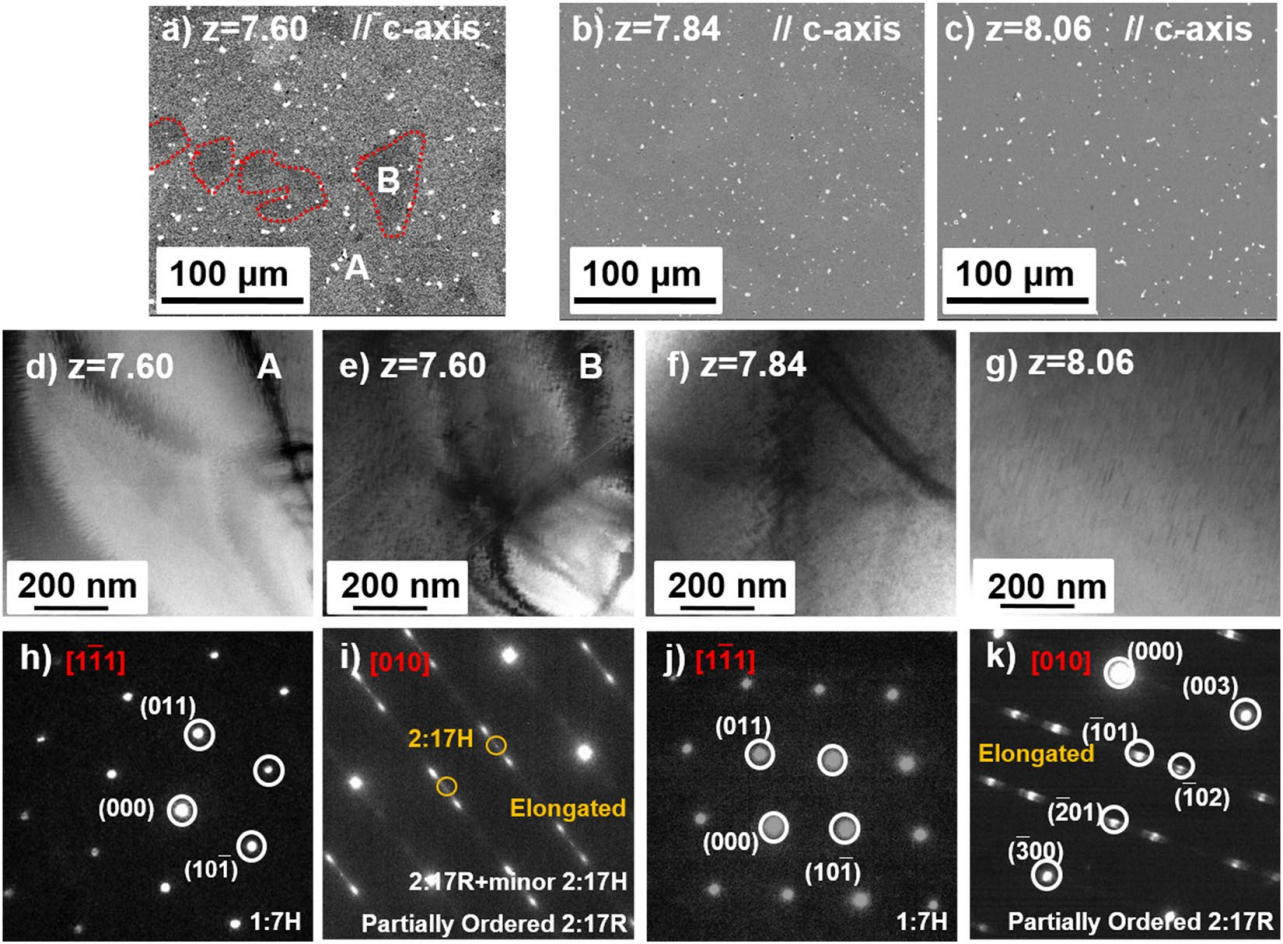
SEM images (**a**–**c**), TEM-BF image of different phases (**d**–**g**) and the corresponding selected area electron diffraction (SAED) patterns (**h**–**k**) of solution-treated Sm(Co_bal._Fe_0.28_Cu_0.053_Zr_0.02_)_z_ (z = 7.60, 7.84, and 8.06) magnets.

**Figure 4:**
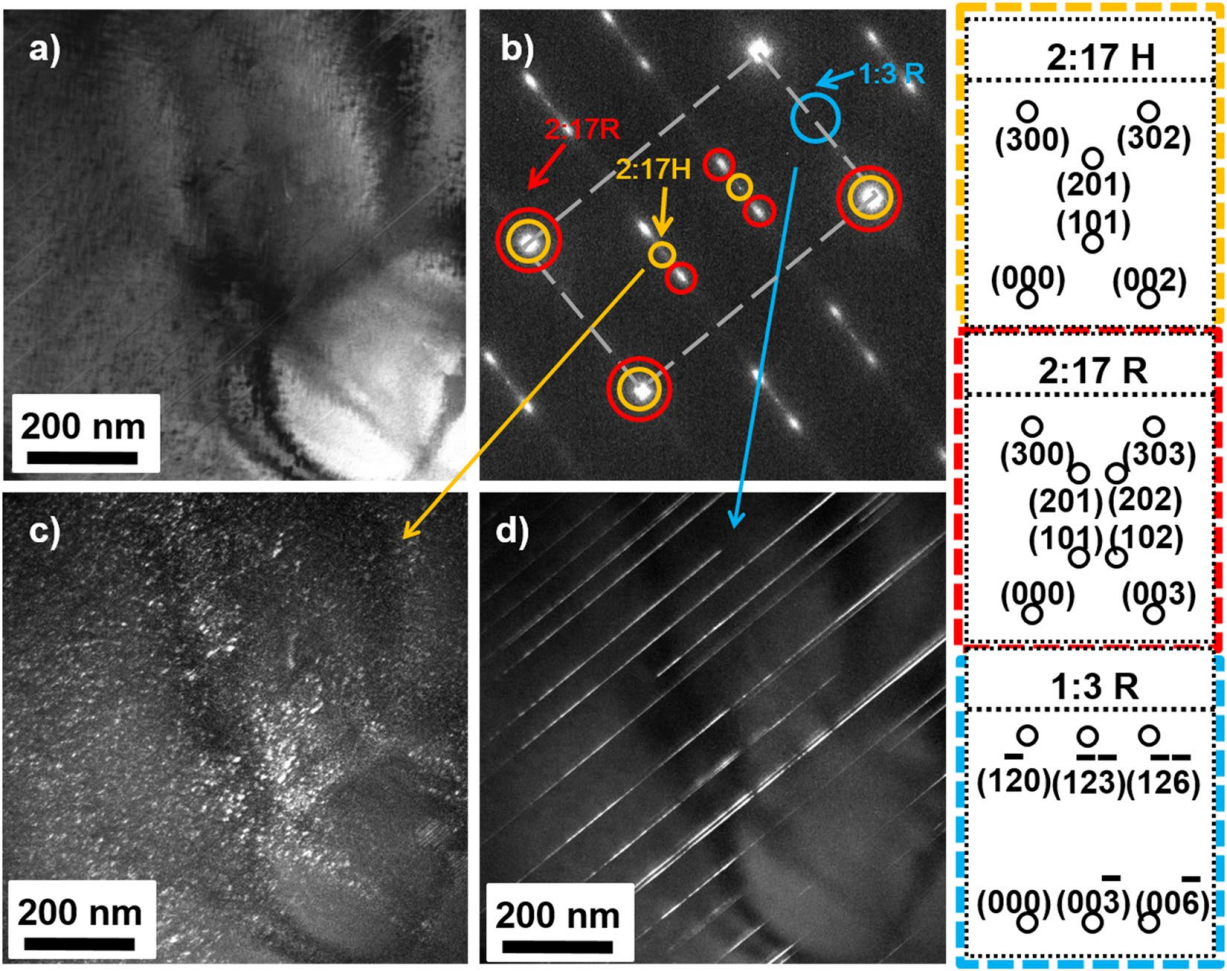
TEM-BF image (**a**), corresponding SAED patterns (**b**) and TEM dark field (TEM-DF) image for the diffraction points of 2:17H (**c**) and 1:3R phase (**d**) of solution-treated Sm(Co_bal._Fe_0.28_Cu_0.053_Zr_0.02_)_7.60_ magnets.

In order to ease the occupational competition between Zr, Fe and Sm and control the main phase of solution precursor, the influence of Sm content to iron-rich Sm(Co,Fe,Cu,Zr)_z_ magnets was investigated. Figure 3 shows the SEM images and corresponding SAED patterns in Sm(Co_bal._Fe_0.28_Cu_0.053_Zr_0.02_)_z_ (z = 7.60, 7.84, and 8.06) solution precursor. For magnets with z = 7.6 (Fig. 3a), apart from the white dots (samarium oxide), the BSE image shows mostly the gray area (marked with A) and part of the black area (marked with B). From the SAED patterns (Fig. 3h,i), it indicates that the area A is a single 1:7H phase. As for the area B, which shows elongated diffraction patterns, consists of 2:17R and minor 2:17H phase (detailed analysis is shown in Fig. 4). As for the magnet with z = 7.84 and z = 8.06, both homogeneous contrast are observed in BSE images (Fig. 3b,c). However, with characterization of SAED patterns, it can be seen that the single 1:7H phase is obtained in magnet with z = 7.84 (Fig. 3j), while only partially ordered 2:17R phase is observed in magnet with z = 8.06 (Fig. 3k).

As for the magnet with z = 7.60, the TEM bright field (TEM-BF) image is taken by tilting the selected grain with its ${[{\rm{0}}{\rm{1}}{\rm{0}}]}_{2:\mathrm{17H}}={[{\rm{0}}{\rm{1}}{\rm{0}}]}_{2:\mathrm{17R}}={[{\rm{2}}{\rm{1}}{\rm{0}}]}_{1:\mathrm{3R}}$ zone axis parallel to the electron beam (Fig. 4a). The correspondence SAED pattern is the diffraction pattern of 2:17R and 2:17H phase, as is shown in Fig. 4b. The elongated spots in SAED of 2:17R indicate that the 2:17R phase is partially ordered. Besides the matrix phase, it is interesting to find lots of strip-like precipitated phases embedded in the matrix phase, which have similar distribution of Zr-rich 1:3R lamellar phase in aged magnets. In order to clarify this phase, the TEM dark field (TEM-DF) image for the key diffraction points of 2:17H and 1:3R phase is shown in Fig. 4c,d. Some white areas are observed and scattered across the matrix phase, suggesting the strip phase is not 2:17H phase. According to the characteristic diffraction point of 1:3R phase in TEM-DF image, the white area is completely consistent with the distribution of strip-like phase in SEM image, which indicates the strip phase to be the 1:3R phase.

**Figure 5:**
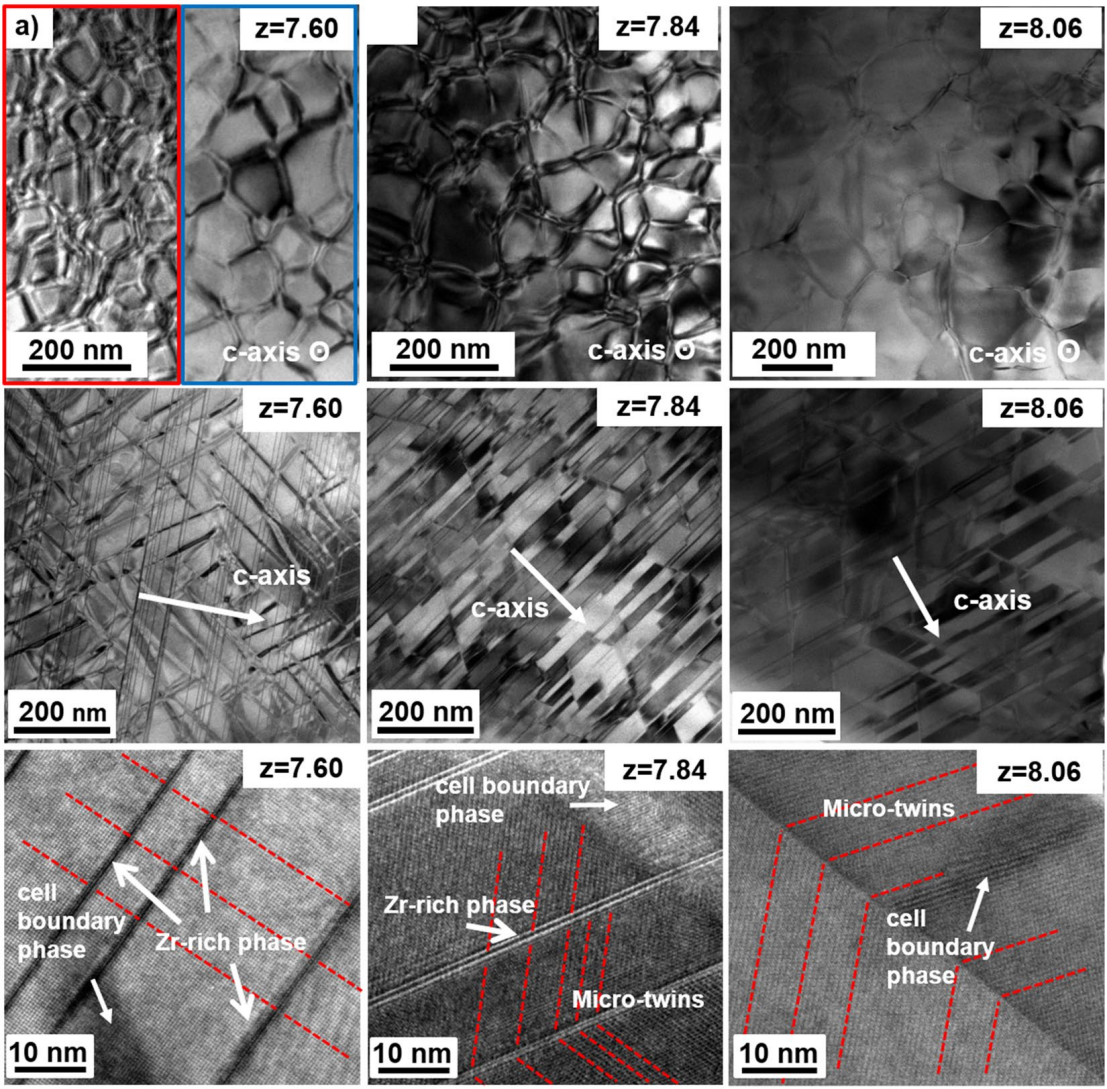
TEM-BF images of aged Sm(Co_bal._Fe_0.28_Cu_0.053_Zr_0.02_)_z_ (z = 7.60, 7.84, and 8.06) magnets with the observation surface perpendicular (**a**–**c**) and parallel (**d**–**f**) to c-axis. Corresponding High-resolution TEM of representative sample region (**g**–**i**).

The microstructure of aged magnets was characterized by TEM-BF images. From the images perpendicular to c-axis, it can be seen that the cellular structure show great difference (Fig. 5a–c). Two morphology features of celluar structure are observed in the magnets with z = 7.60. One shows small and very uneven cellular structure with some cell boundaries cluster, the other shows larger cellular structure with thin and distinct cell boundaries. When z = 7.84, the cell structure becomes more homogeneous. However, as the Sm content further decreases to z = 8.06, the cells grow very large (~200 nm) and the cell boundaries become thin and obscure. According to the images parallel to c-axis, lots of lamellar phases are observed in aged magnets with z = 7.60 and 7.84 while fewer lamellar phases appear with z = 8.06 (Fig. 5d–f). Although there are some lamellar phases remained from the solution-treated magnets with z = 7.60, the density of lamellar phase in aged magnet is obviously higher, which indicates that some of lamellar phases are formed during aging treatment. Besides, more micro-twin structures are observed in aged magnets with z = 8.03 than z = 7.60 and z = 7.84 (Fig. 5g–i). From the cell structure analysis, it can be seen that the cell size and lamellar phase are closely related with the phase structure of solution-treated magnets. The uneven cellular structure that tends to grow up in iron-rich Sm(Co,Fe,Cu,Zr)_z_ magnets has been observed in many research results, which leads to the great decrease of coercivity^18^.

**Figure 6:**
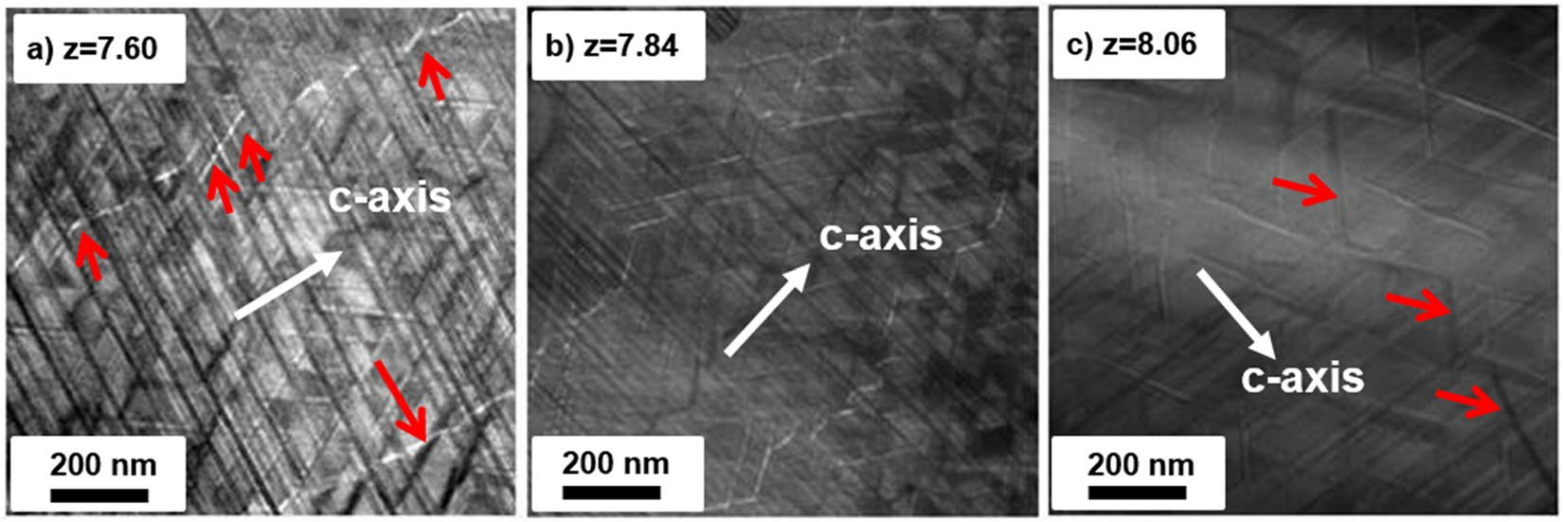
Lorentz-TEM images of aged Sm(Co_bal._Fe_0.28_Cu_0.053_Zr_0.02_)_z_ magnets (**a**) z = 7.60, (**b**) z = 7.84, and (**c**) z = 8.06.

The distribution of magnetic domain wall was characterized by Lorentz-TEM. Clearly, some magnetic domain walls of magnets with z = 7.60 and 8.06 (Fig. 6a,c), marked with red arrows, cross through the cell phase, while the magnet domain walls of magnet with z = 7.84 are well distributed along the cell boundary (Fig. 6b). As we know, the coercivity of 2:17-type Sm-Co is surely related to the pinning of magnetic domain wall in the cell phase or cell boundary phase^23,24^. In z = 7.84 magnet, the high coercivity indicates that the cell boundary phase is the strong pinning center. However, in magnets with incomplete cellular structure, it can be seen that part of magnetic domain wall would go across the cell phase. According to the low coercivity in these magnets, it indicates that the magnetic domain wall that go across cell phase can be easily moved in applied magnetic field.

**Figure 7:**
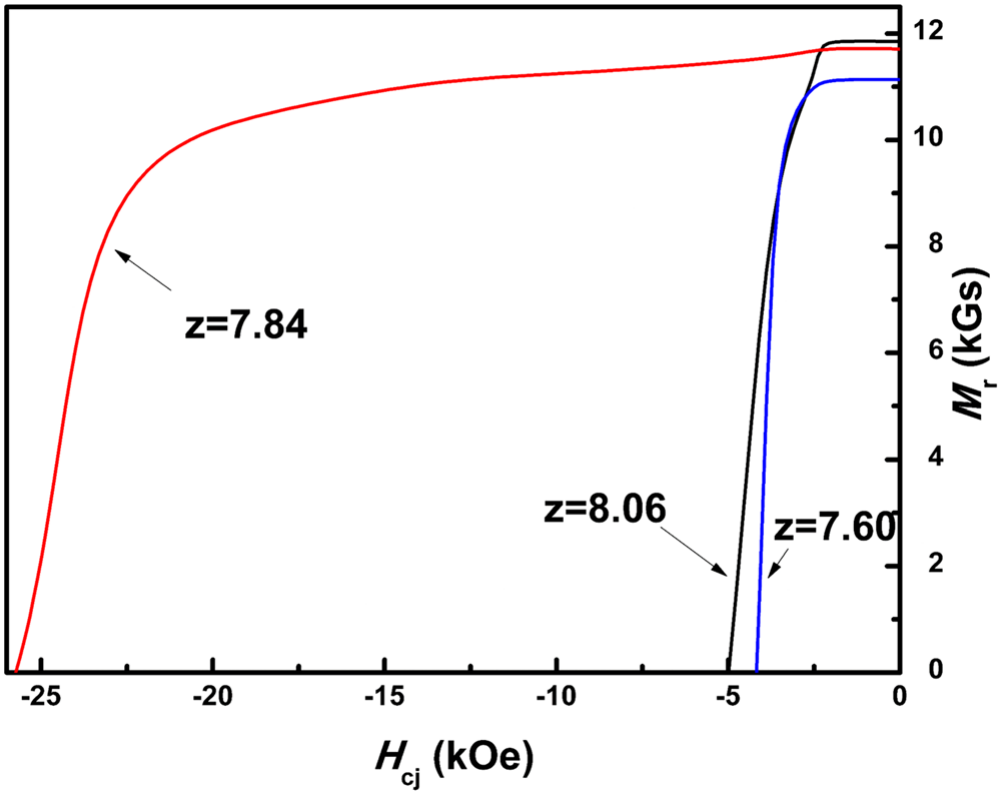
Demagnetization curves of aged Sm(Co_bal._Fe_0.28_Cu_0.053_Zr_0.02_)_z_ (z = 7.60, 7.84, and 8.06) magnets.

The room temperature magnetic properties of aged Sm(Co_bal_Fe_0.28_Cu_0.053_Zr_0.02_)_z_ (z = 7.60, 7.84, and 8.06) magnets were measured. Figure 7 shows the room temperature demagnetization curves of aged Sm(Co_bal._Fe_0.28_Cu_0.053_Zr_0.02_)_z_ (z = 7.60, 7.84, and 8.06) magnets. Table 2 gives the magnetic data of aged Sm(Co_bal_Fe_0.28_Cu_0.053_Zr_0.02_)_z_ (z = 7.60, 7.84, and 8.06) magnets. Clearly, very low coercivity is shown in the magnets with z = 7.60 and 8.06, while high coercivity about 25.7 kOe is obtained in magnets with z = 7.84. It is obviously related to the cellular structure of magnets. The very uneven of cellular structure in magnet with z = 7.60 and the missing lamellar phase in magnet with z = 8.06 lead to the low coercivity. Furthermore, the distribution of magnetic domain wall also indicates that the magnets with z = 7.60 and z = 8.06 are more easily demagnetized. Additionally, with the increase of Sm content, the remanence of Sm(Co_bal_Fe_0.28_Cu_0.053_Zr_0.02_)_z_ (z = 7.60, 7.84, and 8.06) magnets decreases monotonously.

**Table 2:** Magnetic properties of aged Sm(Co_bal._Fe_0.28_Cu_0.053_Zr_0.02_)_z_ (z = 7.60, 7.84, and 8.06) magnets.

	Remanence *B*_r_ (*kGs*)	Coercivity *H*_cj_ (*kOe*)	Maximum Energy Product *(BH)*_max_ (*MGsOe*)
z = 7.60	11.14	4.28	22.74
z = 7.84	11.71	25.70	32.80
z = 8.06	11.85	5.02	22.26

## Discussion

Considering the Zr atomic radius, the Zr atom is larger than Fe, Co and Cu atoms and can substitute for Sm atoms or dumbbell Co atoms in 1:7H phase. According to the results of Ray^25^, Luo *et al*.^26^ and Huang *et al*.^27^, in consider of the decrease in lattice parameter *c*, it suggested that Zr atoms have the preference to occupy dumbbell Co-Co pairs in the form of Zr-Vacancy in Sm_2_Co_17_ compound because of the lower centre distance of Zr-Vacancy, though the substitution for Sm was possible^28^.

In the process of solution treatment for as-cast alloys, the Sm-rich grain boundary phase gradually dissolves into matrix 1:7H phase, which means Sm atoms can recapture 1*a* site and squeezed out Zr atoms to dumbbell Co-Co site (2*e* site). However, Fe atoms also have a strong preference for dumbbell Co-Co sites in 1:7H phase^29^, which would result in the occupational competition of Fe and Zr atoms. Therefore, the increasing Fe content could suppress the Zr preference for dumbbell Co-Co sites and lower solid solubility of Zr in 1:7H phase, which causes the precipitation of Zr-rich 1:3 phase in Sm(Co,Fe,Cu,Zr)_z_ alloys with high content of Fe. As for the main phase, with Sm content remaining unchanged, if the content of Fe is too high, it would reduce the content of Zr in 1:7H phase, which can result in the instability of 1:7H phase and promote the ordering transformation. Thus, the main phase in Sm(Co, Fe, Cu, Zr)_z_ alloys with high content of Fe tend to be partially ordered 2:17R phase. Importantly, the single 1:7H precursor phase is critical to obtain the desired final cellular structure, which determines the final magnetic properties.

In order to ease the occupational competition between Sm, Fe and Zr to stabilize 1:7H and suppress ordering transformation, a feasible method is to adjust the content of Sm. In solution treated Sm(Co_bal._Fe_0.28_Cu_0.053_Zr_0.02_)_z_ (z = 7.60, 7.84, and 8.06) magnets, the main phase structure shows a great change with different Sm contents. A little higher content of Sm (z = 7.60) would make 1:7H phase partially decomposed into partially ordered 2:17R, 2:17H and 1:3R phase, while the single 1:7H phase can be formed with decreasing Sm content (z = 7.84). However, it would change to partially ordered 2:17R completely with further decreasing Sm content (z = 8.06). Although both 1:7H and partially ordered 2:17R can form cellular structure through precipitation hardening process after aging treatment, but they show different sizes and growth behaviors. Compared with 1:7H phase, the cellular structure formed by solution precursor with partially ordered 2:17R, tends to grow up more quickly and have less lamellar phase. Thus the cellular structure is uneven for magnets formed by the mixture phase structure of solution precursor (z = 7.60). With total partially ordered 2:17R phase, the aged cellular structure becomes very large and incomplete. This could explain why the cell size of iron-rich 2:17 type Sm-Co magnets tends to grow up quickly in many other researches^18^.

Generally, the coercivity of Sm(Co,Fe,Cu,Zr)_z_ magnets is related to the difference of magnetocrystalline anisotropy between cell phase and cell boundary phase^23^. The larger of this difference is, the stronger pinning effect can be obtained. According to many existing research results, the pinning center could be cell boundary phase or cell phase, which is related to the distribution of Cu in cell boundary phase^23,30–32^. Actually, it is not the key point in this paper. As we know, all pinning theories should be based on the complete cellular structure. If this structure is incomplete, it would impact the distribution of domain wall, and is hard to analys based on the traditional theory. In our magnets with different Sm content, the cellular structure is very different. According to the coercivity value and the distribution of magnetic domain wall, it indicates that the pinning center in complete cellular structure is cell boundary phase, while the incomplete cellular microstructure, especially the aggregation of cell boundary phase and missing lamellar phase, would result in the magnetic domain walls going across the cell phase. This kind of region should be the area with weakened pinning field.

From the room temperature demagnetization curves (Fig. 7) and magnetic data in Table 2, it could be seen that the remanence increases from 11.14 *kGs* to 11.85 *kGs* with the decreasing Sm content. This trend is illustrated by the result of Matthias *et al*. that less Sm leads to the increased volume fraction of 2:17R phase (main source of the remanence) and decreased volume fraction of 1:5 cell boundary phase^33^. In addition, the maximum energy product is closed to the results of Duerrschnabel *et al*. in the case of lower Fe content in our magnets^24^. Hourich *et al*. have achieved the *(BH)*_max_ of about 35.4 *MGsOe* in magnets with much higher Fe content but have yet to realize the full potential in its coercivity (19.8 *kOe*)^34^. They reported that the impurity phase was the main reason for the incomplete cellular structure and low coercivity. However, in this paper, based on the detailed analysis, we believe that the ordering transformation of the main phase in solution precursor tends to be generated by increasing iron content, resulting in the deterioration of the final cellular structure and the coercivity.

## Conclusion

In this work, we have reported the evolution of phase constitution and microstructure in iron-rich 2:17-type Sm-Co magnets and obtained high magnetic properties with a large coercivity *H*_*c*j_ of 25.70 *kOe* and maximum energy product *(BH)*_max_ of 32.80 *MGOe* by adjusting Sm content at z = 7.84. Based on these researches, two mechanisms have been revealed:

(i) Sm/Cu-rich 1:5 phase and Sm/Cu-rich 2:7 phase in as-cast alloy can be eliminated, while partially ordered 2:17R phase (mixture with Th_2_Zn_17_ and Th_2_Ni_17_ structure) and Zr-rich 1:3 phase tend to appear in alloys with higher Fe content after solution treatment. The formation of the impure phase is related to the solubility and occupation of Zr atoms in 1:7H phase. The increased Fe content could lower the solid solubility of Zr in dumbbells pairs(*2e* site) and inappropriate Sm content would restrain the occupation of Zr at Sm site (*1a* site), which induces the instability of 1:7H phase and promotes the ordering transformation from 1:7H to partially ordered 2:17R and precipitation of lamellar Zr-rich 1:3R phase.
(ii) The ordering transformation could be restrained and single 1:7H could be obtained by modifying Sm content in solution precursor of iron-rich 2:17-type Sm-Co magnets. Different phase in solution precursor evolve into different cellular structures after aging treatment. Solution precursor with single 1:7H tends to form uniform cellular structures and more lamellar phase, leading to a high coercivity and magnetic energy product. However, solution precursors with 1:7H, partially ordered 2:17R, 2:17H and 1:3R tend to evolve into uneven cellular structures, while precursors with partially ordered 2:17R phase tend to form larger cellular structures and less lamellar phase, thus both poor magnetic properties.

Above results indicate that if the ordering transformation can be effectively restrained in solution-treated 2:17-type Sm-Co magnets with higher content of Fe, much higher magnetic energy product could be achieved.

## Method

### Sample synthesis

The Sm(Co_bal._Fe_x_Cu_0.053_Zr_0.02_)_7.84_ (x = 0.25, 0.30, 0.35, and 0.40) and Sm(Co_bal._Fe_0.28_Cu_0.053_ Zr_0.02_)_7.84_ (z = 7.60, 7.84, and 8.06) ingots were prepared by induction melting under argon atmosphere. Excess Sm of about 3% was added to compensate the Sm losses.

The Sm(Co_bal._Fe_x_Cu_0.053_Zr_0.02_)_7.84_ (x = 0.25, 0.30, 0.35, and 0.40) ingots were subjected to solid solution treatment in argon atmosphere at 1120 °C–1200 °C for 3H followed by rapid cooling to room temperature.

The Sm(Co_bal._Fe_0.28_Cu_0.053_Zr_0.02_)_7.84_ (z = 7.60, 7.84, and 8.06) as-cast alloys were pre-crushed into coarse powders and jet-milled into 3.8 μm via jet-milling (JM). The fine powders were oriented in a magnetic field of 2T and then pressed isostatically at 160 MPa. The green compacts were sintered at 1200 °C for 30 min, homogenized at 1175 °C for 3H, and quenched to room temperature. The subsequent isothermal aging was at 830 °C for 16H, followed by slow cooling (0.7 °C/min) to 400 °C, holding for 4H, and finally rapid cooling to room temperature.

### Measurement and characterization

The peak ratio analysis was carried out by X-ray diffraction (XRD) with Cu *K*_*a*_ radiation. A FEI Quanta FEG 250 scanning electron microscope (SEM) equipped with an energy dispersive X-ray spectroscopy (EDX) detector were used on the alloys. For the SEM studies, the samples were demagnetized, grinned and polished with the hexagonal c-axis of Sm_2_Co_17_ lying perpendicular to the polishing direction. Transmission electron microscope (TEM) observations were performed using Tecnai F20 system with an acceleration voltage of 200 kV to determine the microstructure on the nanometer scale. The acquisition time is 0.2 s and 1.2 s for morphology and selected-area diffraction analysis, respectively. The spot size is 1. Lorentz microscopy observations were performed using JEM-2100F with Fresnel mode to study the distribution of magnetic domain wall. For the TEM and Lorentz microscopy studies, the samples were demagnetized, thinned via conventional grinding and polishing plane to a thickness of ~50 μm with the hexagonal c-axis of Sm_2_Co_17_ lying parallel and perpendicular to the polishing direction, then using Ar ion milling and finally mounted on a Cu hoop. The demagnetization curves were measured using a closed circuit B-H apparatus (NIM 500C) after pre-magnetized in a pulsed field of 80 *kOe*.

The detailed overall process can be found as Supplementary Fig. S1.

## Electronic supplementary material

Supplementary Information
