# Supplementary material for: The evolution of phase constitution and microstructure in iron-rich 2:17-type Sm-Co magnets with high magnetic performance
Source: Sci Rep. 2018 Jun 14;8:9103. doi: 10.1038/s41598-018-27487-x (PMC6002360; doi:10.1038/s41598-018-27487-x)
Supplement: Supplementary file 1 — Supplementary Information [file 41598_2018_27487_MOESM1_ESM.pdf]

The evolution of phase constitution and microstructure in iron-rich 2:17-type Sm-Co magnets with high magnetic performance

Chaoyue Zhang[1,2] , Zhuang Liu[1,*], Ming Li[1], Lei Liu[1], Tianyi Li[1], Renjie Chen[1], Don. Lee[1,3], A Ru Yan[1,*]

[1] Key Laboratory of Magnetic Materials and Devices, Ningbo Institute of Material Technology and Engineering, CAS, Ningbo 315201, China

[2] University of Chinese Academy of Sciences, Beijing 100049, China

[3] University of Dayton, Dayton, OH, USA

Corresponding Authors: Z.L. (email: zliu@nimte.ac.cn) and A.Y. (email: aruyan@nimte.ac.cn).

The schematic of the overall process

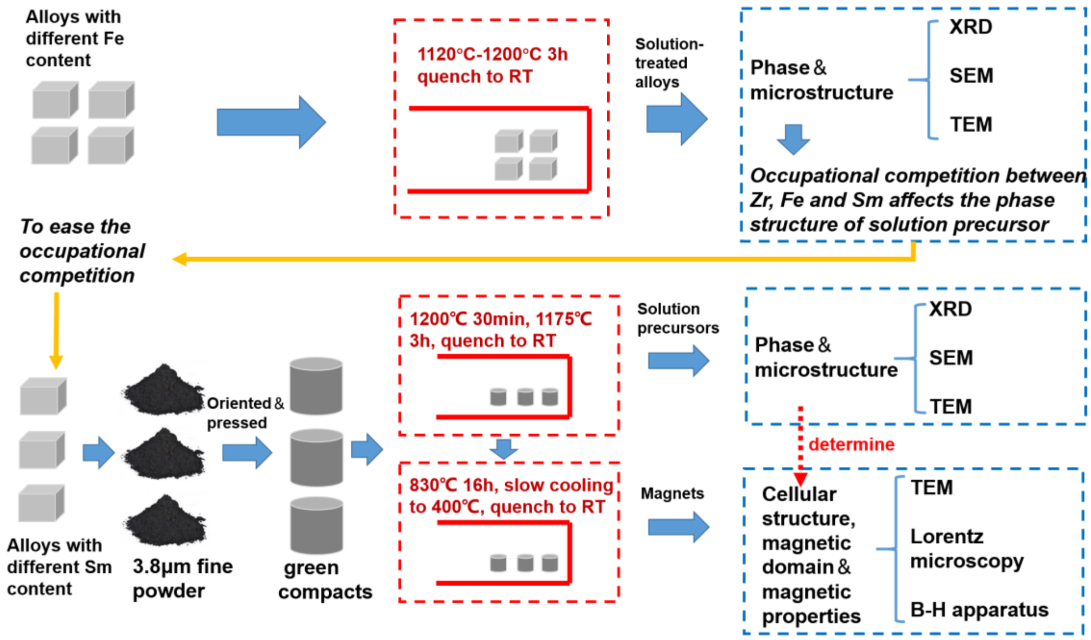

Supplementary Figure S1. The detailed overall process of the experiment.
